# Parkinson’s Disease: A Complex Interplay of Mitochondrial DNA Alterations and Oxidative Stress

**DOI:** 10.3390/ijms14022388

**Published:** 2013-01-24

**Authors:** Sarah Ciccone, Emiliano Maiani, Giovanna Bellusci, Marc Diederich, Stefania Gonfloni

**Affiliations:** 1 Department of Biology, University of Rome “Tor Vergata”, Via della Ricerca Scientifica, 00133 Rome, Italy; E-Mails: sarah.ciccone@gmail.com (S.C.); emiliano.maiani@gmail.com (E.M.); grisolia1982@libero.it (G.B.); 2 Laboratoire de Biologie Moléculaire et Cellulaire du Cancer, Kirchberg Hospital, 9 Rue Edward Steichen, 2540 Luxembourg, Luxembourg; E-Mail: marc.diederich@lbmcc.lu; 3 College of Pharmacy, Seoul National University, 599 Gwanak-ro, Gwanak-gu, Seoul 151-742, Korea

**Keywords:** neurodegenerative diseases, Parkinson’s disease (PD), base excision repair (BER), mitochondria, oxidative stress, reactive oxidative species (ROS), reactive nitrogen species (RNS), c-Abl, reduced glutathione (GSH), oxidized glutathione (GSS-)

## Abstract

Parkinson’s disease (PD) is one of the most common age-related neurodegenerative diseases. This pathology causes a significant loss of dopaminergic neurons in the *Substantia Nigra*. Several reports have claimed a role of defective nuclear and mitochondrial DNA repair pathways in PD etiology, in particular, of the Base Excision Repair (BER) system. In addition, recent findings, related to PD progression, indicate that oxidative stress pathways involving c-Abl and GST could also be implicated in this pathology. This review focuses on recently described networks most likely involved in an integrated manner in the course of PD.

## 1. Introduction

Over recent years considerable research efforts have focused on age-related neurological disorders. An emerging theme is that many neurodegenerative diseases are most often associated with altered DNA repair mechanisms (*i.e*., Base Excision Repair (BER), Double Strand Break (DSB) and Single Strand Break (SSB) Repair) [1–3], mitochondrial defects and oxidative stress [4].

At first glance, identification of the symptoms at an early stage of neurodegenerative diseases is complex due to their similarity with typical aging [5]. Moreover, only post-mortem studies can be conducted on patients affected by these pathologies. The complexity of the neurodegenerative diseases is linked to the structural characteristic of the Central Nervous System (CNS), which is composed of brain and spinal cord. In these energy-demanding organs, oxidative damage is very frequent, caused by a sustained oxygen consumption coupled with an inefficient anti-oxidant defense system [6]. Convincing evidence indicates that oxidative stress, mitochondrial dysfunction and accumulation of mutations in mitochondrial DNA (mtDNA) are hallmarks of neurodegenerative disorder progression [7] and of aging [8–10]. MtDNA is more susceptible to mutations compared to nuclear DNA (nDNA) as it is more exposed to damaging events caused by Reactive Oxygen Species (ROS) generation. Persistent ROS exposure, lack of protection by histone, and reduced DNA repair pathways may lead to harmful point mutations or large-scale rearrangements of mtDNA (reviewed by [7] and references within). This ends in a progressive accumulation of mtDNA mutations with age, in particular, in tissues with active oxidative metabolism such as brain [11]. Of note, mtDNA alterations could potentially impact on enzymes involved in ATP production fostering ROS generation. In return, this may cause neuronal cell death, altering both morphology and function of the brain [6,12,13]. In line with this, several reports have correlated accumulation of mtDNA mutations with increased oxidative stress and mitochondrial dysfunction in aging [8,9].

Recent reports show that the non-receptor tyrosine kinase c-Abl is involved in neurodegenerative disease progression [14]. Of note, c-Abl can interact with various proteins implicated either in DNA repair [15–17], or in oxidative stress response and even can play a positive role in autophagy, the latter being a process associated with neuroprotection [18,19]. In this review, we focus on principal mechanisms that lead to neurodegenerative etiology, starting by the most frequently cited DNA repair pathways (like BER), and also including the emerging role of mitochondrial and oxidative stress. We choose one of the best-known neurodegenerative diseases, Parkinson’s disease (PD), as a paradigm, for assembling each single signaling cascade into an integrated scenario. A deep knowledge of the interplay between mitochondrial alterations and redox signaling may help us to identify the signaling circuitry underlying age-related neurodegenerative diseases. Mitochondrial genome instability and oxidative stress most likely behave as “synthetic lethal interactions” for death decisions in neurons. In turn, this could be exploited to design new combined therapeutic strategies for PD patients.

## 2. DNA Damage and Repair

DNA integrity plays a central role in normal cell growth and the serious consequences deriving from DNA damage are quite intuitive. Persistent DNA damage, in fact, underlies the most important and common human diseases including neurodegenerative diseases and cancer [20]. DNA damage can be caused by endogenous (like ROS) or exogenous (*i.e.*, ionizing radiation and pesticides) sources. Briefly, the principal types of DNA modifications include (a) apyrimidinic site (loss of a base due to a *N*-glycosil bond cleavage) [21], (b) deamination (loss of an amino group) [22], (c) cyclobutane pyrimidine dimers, (one of the principal consequences of UV radiation, in which two pyrimidines situated on the same DNA strand are covalently linked) [22], (d) trinucleotide repeats expansion (particularly frequent in neurodegenerative diseases) [23], (e) single strand breaks (SSBs) (a very frequent kind of DNA damage) [24] or a double strand break (DSB) [1]. A strand break is a particularly aggressive type of DNA damage eventually leading to replication fork collapse [25]. DNA strand breaks are the main types of damage present in the neurodegenerative diseases and in aging [26].

In neurons, prolonged oxygen consumption fosters ROS production. Constant ROS generation may cause DNA damage in the mitochondria thus promoting mitochondrial dysfunctions. Increased ROS production can also cause constant oxidative (DNA) damage in the nucleus. The causal relationship between ROS and mitochondrial (or nuclear) DNA damage strengthen the concept that maintaining ROS at the physiological level is essential for neuronal homeostasis.

When DNA damage occurs, some sensors are activated to induce DNA damage response (DDR). Among them are ATM (ataxia telangiectasia mutated), ATR (ATM and Rad3 related) and DNAPK (DNA dependent protein kinase) kinases belonging to the phosphoinositide-3-kinase-like kinase family. ATM/ATR/DNAPK can lead the cell to DNA repair and cell cycle arrest or apoptosis, if DNA lesions are irreparable [27,28]. According to the type of DNA lesions, cells use distinct mechanisms of DNA repair [29]. Among them, Nucleotide Excision Repair (NER), Base Excision Repair (BER), Mismatch Repair (MMR) and Double Strand Breaks (DSBs) repair, the latter includes two distinct mechanisms based on Homologous Recombination (HR) and Non Homologous End Joining (NHEJ) [1,5,29]. Briefly, DSBs induce a signaling cascade mediated by Rad50-MRE2-NSBI (MRN-complex) and by ATM leading to DNA repair. The choice between HR and NHEJ is tissue specific and depends on the cell cycle phase. HR is active in late S-G2 phases, whereas the NHEJ system acts in G1-early S phase. In the NHEJ pathway, the heterodimer protein KU binds to DSBs, activating DNAPK, which performs end processing. Furthermore, a DNA polymerase promotes repair and synthesis and the pathway ends through the activity of DNA ligase (LIG4/XRCC4). On the contrary, in the HR pathway, the intact chromosome is used like a template to repair the damaged strand. In detail, the DNA ends are processed by the activity of the MRN complex with the help of BRCA1 (Breast Cancer 1), whereas RAD51 monomers invade the damaged strands producing nucleoprotein filaments formation. Further steps lead to the formation of a displacement loop (D-Loop) and DNA repair synthesis mediated by DNA polymerase, with the formation of an intermediate Holliday junction. At the end, DNA ligase LIG1 joins the ends also through the activities of some helicases (Bloom Syndrome RecQ Helicase BLMRecQ, Topoisomerase III alpha TOPO3α and Bloom Syndrome associated protein of 75 kDa BLAP75) that release the Holliday junction [26,30].

NER repair is promptly engaged when UV light and polycyclic aromatic hydrocarbons induce alteration of the DNA helix structure. This pathway can be separated into two different cascades namely Global Genome NER (GGNER) and Transcriptional-coupled NER (TCNER). In GGNER, the XPC-HR23B-CEN2 complex (Xeroderma pigmentosum, complementation group C- RAD23 homolog B- centrin, EF-hand protein, 2 complex), with the help of DNA Damage Binding (DDB) complex, senses the damage and recruits the transcription factor TFIIH. In TCNER, damage is recognized by DNA polymerase II with the help of the proteins CSB, CSA (Cockayne Syndrome B Protein and Cockayne Syndrome A Protein) and XAB2 (XPA binding Protein 2). TFIIH is also recruited, and from this point, both GGNER and TCNER proceed similarly. TFIIH uncoils the DNA region in the proximity of the lesion and opens the damaged double helix together with XPB (Xeroderma Pigmentosum complementation group B) and XPD (Xeroderma Pigmentosum complementation group D). Later, the replication protein A (RPA) links the opened DNA to the undamaged strand and endonucleases XPF (Xeroderma Pigmentosum complementation group F) and XPG (Xeroderma Pigmentosum complementation group G) incise the damaged DNA 5′ and 3′ removing the damage and leading to a single strand gap. At this point DNA polymerase δ/κ/ɛ fill the gap and a DNA ligase (LIG1 (Ligase 1 DNA ATP-dependent) or LIG3α-XRCC1 (Ligase III DNA ATP-dependent, X-ray repair complementing defective repair in Chinese hamster cells 1) links the DNA backbone [31–33]. In the brain, the principal DNA repair pathway is BER, probably because it is the major pathway used for oxidative damage repair. Monofunctional or bifunctional glycosylases mediate the excision of the damaged base, leading to the formation of an abasic site (AP site). Furthermore, DNA polymerase β and, two enzymes APE1 (AP endonuclease 1) and PNK1 (Polynucleotide Kinase 3′-phosphatase) perform end processing, leading to a 3′-OH and 5′-P termini. At this point, two different kinds of BER can occur: a short-patch BER (SPBER) or, when the numbers of nucleotides replaced is between 2 and 13, a long patch BER (LPBER). In SPBER, DNA repair is conduced by Polβ with the help of XRCC1. Lastly, DNA ligation is made by LIG3. In LPBER, DNA repair and synthesis are performed by Pol δ/ɛ also with the participation of PCNA (Proliferating Cell Nuclear Antigen) and RFC (Replication Factor C), both factors lead to the formation of 5′flap that is removed by FEN1 (flap structure-specific endonuclease 1). The final step is performed by DNA ligase LIG1 [34,35].

MMR is another evolutionary conserved mechanism of DNA repair. MMR is implicated in repairing base-base mismatch, or in removing insertion loop arising during replication and recombination [1,36,37]. In this mechanism, MSH (MutS homolog) proteins interact with each other forming specific heterodimers named MUTSα (formed by MSH2 and MSH6 dimers) and MUTSβ (MSH2-MSH3) that recognize, respectively, base mispairing and base deletion on DNA strands. MUTS dimers, after PCNA binding [38] can recruit MUTL (MutL homolog) dimers, inducing the replacement of damaged region through the action of DNA Polimerase δ and DNA Ligase I [39]. In humans, MMR is very complex and specific protein-complexes recruitment depends on the location of DNA ends with respect to the mismatch [40].

**Figure 1:**
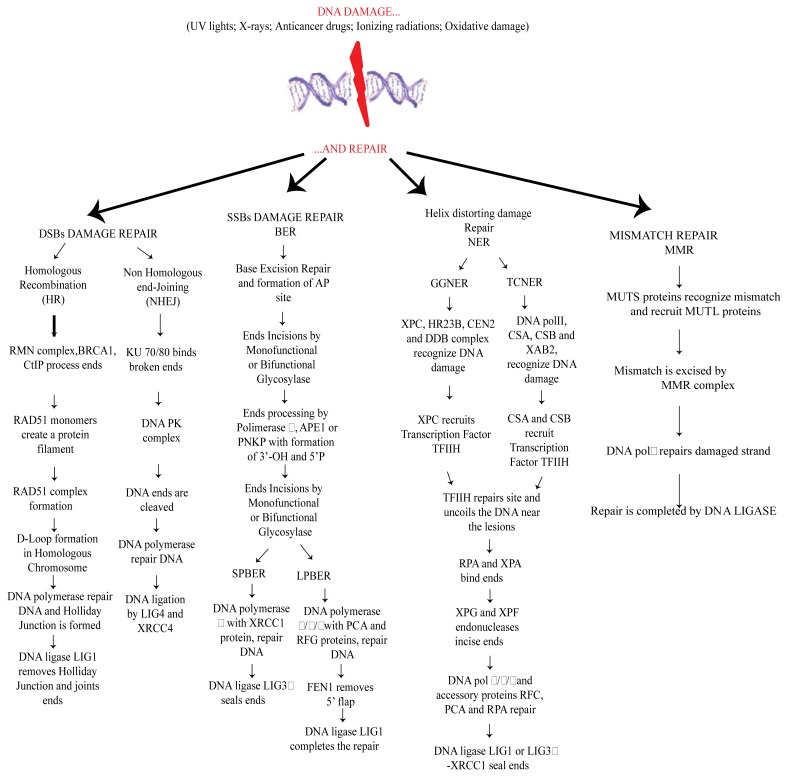
DNA Repair pathways: Homologous Recombination (HR) and Non Homologous Ends Joining (NHEJ) mechanisms are induced by DSBs. Base Excision Repair (BER), Nucleotide Excision Repair (NER) pathways: Global Genome NER (GGNER) and Transcriptional-coupled NER (TCNER) and Mismatch Repair (MMR).

Of note ROS damage can induce nucleic acid breakage and also enzyme inactivation, potentially affecting all the components of DNA repair machinery (Figure 1).

## 3. Mitochondrial DNA Alterations in Diseases

As mentioned above, damaged mitochondria play a very important role in the insurgence of several pathologies [41], including neurodegenerative diseases, due to their involvement in ATP generation [10]. According to the endosymbiotic theory, mitochondria were bacteria-like organisms embodied by the host cells during the evolution. These subcellular organelles consist of an outer membrane, an intermembrane space and an inner membrane with typical cristae. The inner membrane contains a multi protein complex of enzymes involved in electron transport and ATP generation [42]. Many groups have focused in recent years on mitochondria, either for the characteristics mentioned above, or because the mtDNA is exclusively transmitted from the mother. Mitochondria are equipped with their own genome consisting of a circular double strand DNA (mtDNA) of 17 kb (see http://www.mitomap). MtDNA encodes only for 13 proteins which are part of the electron transport chain and are involved in the mitochondrial protein synthesis. Mutations affecting mtDNA can cause serious consequences in cells, like neurons, requiring a lot of energy to carry out their functions. Impairment of ATP production is linked to the onset of typical neurological symptoms [43,44]. MtDNA is particularly vulnerable to oxidative damage compared to nDNA, due to its proximity to the electron transport chain. More than 150 mtDNA mutations, leading to pathological phenotypes, have been identified up to now. Most of these pathologies can affect the nervous system, endocrine system, skeletal muscle and heart, and also other body organs (*i.e.*, eyes, kidney, brain and liver) [45,46]. Lethal mutations are normally eliminated in the mammalian ovary, while milder mutations are transmitted to the germline so ensuring genetic variation in the population [47]. Some examples of diseases induced by mtDNA mutations are CPEO (Chronic Progressive External Ophthalmoplegia) [48], KSS (Kearns-Sayre Syndrome) [49], LHON (Leber’s Hereditary Optic Neuropathy) [50], MELAS (Mitochondrial Encephalomyopathy Lactic Acidosis and Stroke-Like Episodes) [51] and NARP (Neurogenic Muscle Weakness, Ataxia and Retinitis Pigmentosa) [52]. Well-known mtDNA mutations identified up to now are: (1) m.3243A < GMTTL, principally responsible of typical symptoms such as diabetes, myopathy, deafness [53]. (2) m.8344A < GMTTK a mutation leading to the appearance of the myoclonic epilepsy ragged red fibers (MERRF) insurgence [54]. (3) Single large-scale mtDNA deletions, present in many neurological disorders, often characterized by reduction of life span in the patients affected by these diseases (*i.e.*, Kearns Sayre syndrome, KSS) ([10] and references within [42,55]). Recently, many groups have found that random mutations in mtDNA can affect lifespan in mice and are associated with premature aging [56,57]. However, only a limited number of mtDNA mutations occur in natural aging [58–63]. Cells adopt several strategies to reduce the effect of mutations occurring into mtDNA. One strategy is mediated by proteases, removing the damaged mitochondrial proteins. Removal of mitochondrial outer membrane proteins is also promoted through ubiquitin-dependent pathways. Another strategy for “mitochondria clearance” is mediated either through the induction of transcription of chaperones, triggered by the presence of unfolded proteins, or eventually through the elimination of entire mitochondrion by autophagy (mitophagy) [64]. The latter seems to be related to mitochondrial fission and fusion processes. These two mechanisms depend on oxidative phosphorylation and membrane polarization of mitochondria [65].

## 4. Mitochondrial DNA Damage Repair

In the last two decades many groups have focused on mitochondrial DNA damage repair. Albeit several studies are still in progress, mitochondria seem to share some DNA repair pathways previously described for nDNA. While the presence of NER pathway in mitochondria has not yet been clarified, emerging evidence reveals the existence of mismatch repair, MMR, and base excision repair, BER [66,67]. Oxidative DNA damage repair is the major pathway observed in PD and the best-known DNA repair system studied in mitochondria. BER consists of different steps starting from base recognition through DNA glycosylases, while DNA repair is completed through a specific DNA ligase. In the mitochondria the mechanism is the same as well as with the enzymes involved in the process. Firstly, DNA damage is recognized by one of two glycosylases, 8-oxoguanine DNA glycosylase-1 (Ogg1) and Uracil-DNA glycosylase (UNG). Both enzymes are also involved in nDNA damage repair, although, they are expressed as splice isoforms into mitochondria [1,68]. Ogg1 is a bifunctional DNA glycosylase that recognizes and cuts 8-hydroxy-guanine. Then, APE1 (the same enzyme implicated in nuclear BER pathway) processes the AP site and leaves DNA Polymerase γ (the only polymerase known in mammalian mitochondria) to insert the correct oligonucleotide (or more oligonucleotides depending on the choice between short-patch BER or long patch BER). Interestingly, recent reports also suggest that APE1 is expressed in mitochondria as a truncated isoform lacking the N-terminal region [69]. The final step is performed by DNA Ligase III, the latter is a splice variant of the LIG3 gene encoding both for nuclear and mitochondrial enzymes. DNA Ligase III is the only DNA ligase detected in mammalian mitochondria [67,70–72].

## 5. Mitochondrial Defects and Oxidative Stress

Mitochondria play a fundamental role in neurodegenerative disorders [68] and in aging [73]. CNS has an urgent need of energy mainly for impulse transmission. For this reason mitochondria are highly enriched in neuronal axons, where the ATP demand is very high. A defect in mitochondrial function, leading to an impaired respiratory chain mechanism, promotes neuronal cell death due to oxidative stress and formation of proteins aggregates (like α-synuclein fibrils in Parkinson’s disease and αβ fibrils in Alzheimer’s disease) [74].

**Figure 2:**
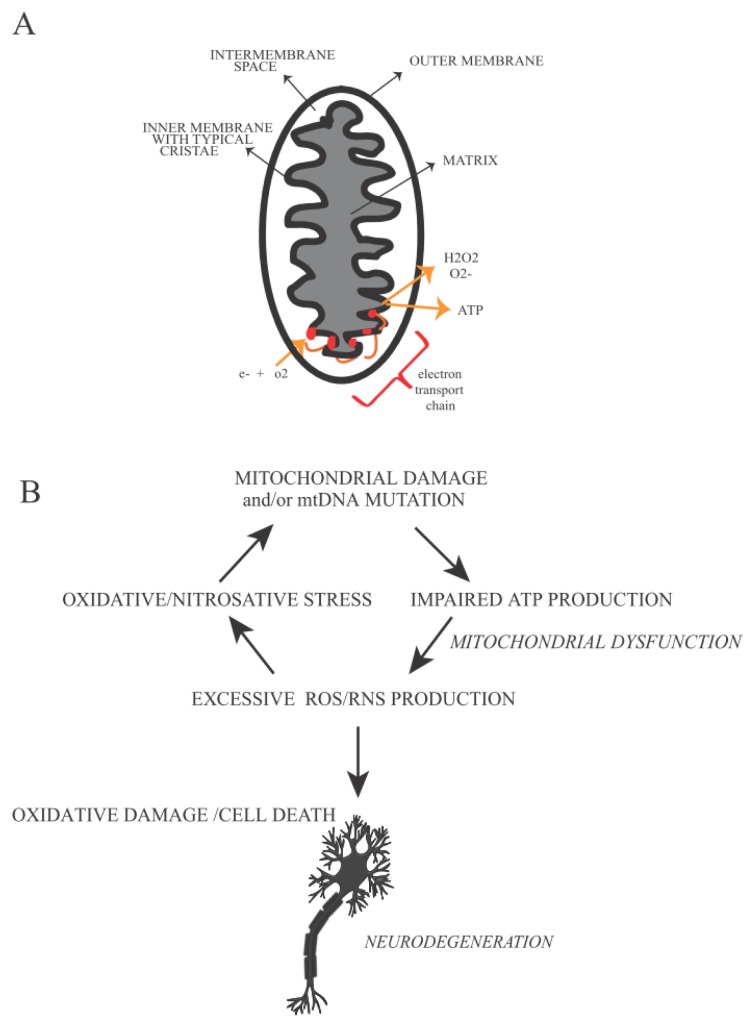
Mitochondria defects in Neurodegenerative Disease: (**A**) Mitochondria are organelles implicated in energy production and endogenous ROS production. They are responsible for more than 90% of the ROS production in the cell. The five proteins of electron transport chain are located in the inner membrane. (**B**) Mitochondrial impairment causes an excessive quantity of ROS/RNS, inducing oxidative stress. The latter is the most frequent event associated with neuronal loss in neurodegenerative diseases.

MtDNA damage impairs mitochondrial energetic capacities, influencing ROS production and eventually leads cells to apoptosis. Both effects are dramatically linked with neurodegenerative disease insurgence and progression [74,75]. Mitochondria represent the principal source of ATP in the cell. During ATP production high amounts of ROS (like superoxide anion, O_2_, hydrogen peroxide, H_2_O_2_ and hydroxyl radicals, •OH) and RNS (for example nitric oxide, NO and peroxynitrite, ONOO-) can be produced. However, if ROS/RNS are generated within a physiological range, this leads to a signaling pathway inducing transcription of antioxidant enzymes, (such as superoxide dismutase (SOD) that converts superoxide into O_2_ into H_2_O_2_, catalase and glutathione peroxidase that reduce H_2_O_2_, glutathione *S*-transferase, heme oxygenase, thioredoxin, glutathione peroxidase *etc.*). Genes encoding these enzymes contain Antioxidant Responsive Element (ARE) activated by redox-sensitive transcription factors (like APE1, Nrf1 and Nrf2 (nuclear factor (erythroid-derived 2)-like 1/2)), which are normally inactivated in the cytosol by specific inhibitors. ROS, RNS and products of lipid oxidation can promote the dissociation of protein inhibitors and transcription factors inducing the synthesis of antioxidant enzymes, phase II detoxification enzymes and stress response proteins [73,76–82]. When the balance between ROS/RNS production and antioxidant enzymes activity is impaired, oxidative damage occurs in the cell producing 8-OHdG (8-hydroxy-2′-deoxyguanosine) and 8-OHG (8-hydroxy-guanosine). The latter are typical markers of oxidative stress observed in the aged human brain in association with synaptic loss and neuronal cell death (Figure 2) [2].

**Figure 3:**
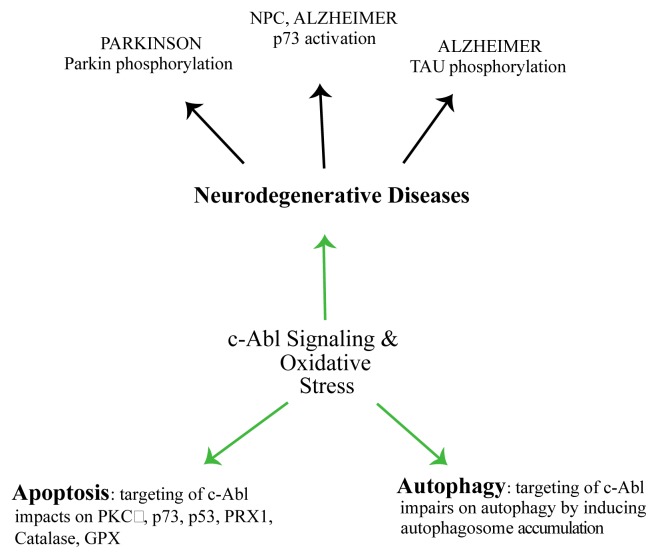
c-Abl signaling and neuronal diseases: c-Abl plays a central role in several pathways and in oxidative stress response. c-Abl modulates cell death by interacting with p73/p53 transcription factors. c-Abl is also involved in molecular mechanisms underlying several neurodegenerative diseases like PD and AD, promoting Parkin and Tau tyrosine phosphorylation.

The mechanisms induced by oxidative stress are very complex and involve several proteins/regulators in addition to the enzymes mentioned above. Among them, a family of non-receptor tyrosine kinases that includes c-Abl (Abl and Abl1) and Abl related genes (Arg and Abl2). c-Abl was early identified as the mammalian homolog of the oncogenic gene product of Abelson murine leukemia virus [83]. c-Abl is involved in a large number of cellular processes, in DNA repair [84] and also in neuronal development and in neurodegenerative diseases [14,18] (Figure 3). In the brain of Alzheimer’s patients c-Abl co-localizes with granuvacuolar degeneration (GVD) and amyloid β fibrils (αβ fibrils). Moreover, these studies also indicate that oxidative stress induces αβ fibril formation in neuronal cells, while over-expression of c-Abl and p73 leads neurons to apoptosis. Interestingly, the c-Abl/p73 pathway is also described in the cerebellum of mice affected by Niemann-Picktype C, a neurodegenerative disease characterized by neuronal loss due to the excessive uptake of cholesterol [19,85–89].

Further studies indicate a coordinated work between c-Abl and Cdk5 (cyclin-dependent kinase 5) in human neuroblastoma (SHSY5Y) cells. Following oxidative stress, c-Abl phosphorylates Cdk5 on Y15, in return, Cdk5 promotes p53 accumulation and neuronal cell death [90].

Another important source of oxidative stress is caused by an excessive accumulation of transition metals. In line with this, Fe and Cu are implicated in the progression of several neurodegenerative disorders. Metal storage proteins, like ferritin and cerruloplasmin, are involved in the imbalance of the rate of generation and sequestration of the transition metals. Of note, these proteins are down-regulated in some neurodegenerative diseases such as PD. Moreover, Cu, Fe and Zn, seem to be increased in senile plaques of AD patients, likely inducing oxidative stress in the brain of AD patients (see [91] and references within). Often oxidative DNA damage is also coupled with a reduced function of DNA repair.

One of the most important antioxidant defense systems relies on GSH detoxification through a non-enzymatic reaction. In the brain, the astrocytes release GSH, providing GSH precursor to neurons. This event regulates GSH metabolism and contributes by improving the neuronal antioxidant defense. Alteration of GSH metabolism has been observed in AD and PD. In particular, GSH reduction can affect either the activity of E1 ubiquitin-ligase and proteasome degradation or JNK-mediated pathway, as a consequence of enhanced oxidative stress [92–94].

All these observations, including the role of oxidative stress, of c-Abl and of GSH in the insurgence and progression of neurodegenerative diseases, suggest a possible participation of other enzymes in this mechanism such as Glutathione *S*-transferases (GSTs). These dimeric proteins are mainly involved in oxidative defense system, and have been classified into nine distinct gene families, ubiquitously expressed in the organism. Each monomer contains a ligand site for GSH (G-site) that is highly conserved in all isoforms, and a site for the binding of electrophilic compounds (H-site) that confers to every class of protein a specific substrate affinity. The reaction catalyzed by GSTs enzymes transforms the electrophilic compounds (both endogenous and exogenous) into more hydrophilic ones after their binding with oxidized GSH (GSS-). Then cells can eliminate these conjugates. Moreover, GSTs are only catalytically active as dimers. Under normal conditions, they are present in the cell as a monomeric pool bound to JNK. This implies that GSTs also play an active role in the regulation of JNK-mediated pathway [95–98]. In line with our hypothesis, recent reports propose GSTs as a component of signaling pathways that induce neuronal loss in neurodegenerative diseases like PD [99].

## 6. Parkinson’s Disease

### 6.1. Progression and Typical Symptoms

**Figure 4:**
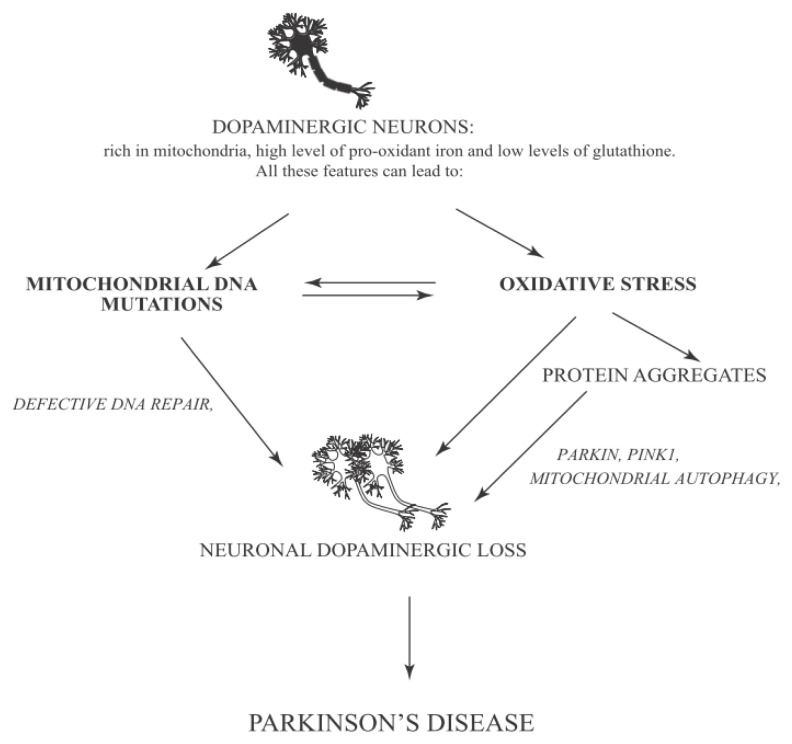
Parkinson’s Disease network: PD is a neurodegenerative disease that affects neuronal cells located in the S*ubstantia Nigra*. PD symptoms are caused by cooperative action of various causes leading to neuronal loss of this area. One of the principal causes of PD progression, as indicated by Braak’s theory, consists of progressive formation of α-syn aggregates and their accumulation in SN *pars compacta*. The figure illustrates also the importance of mitochondria, which are responsible for ATP generation and oxidative stress. PINK1 is localized in the inner membrane of mitochondria (see text). However, under pathological conditions, PINK1 moves to the outer membrane and recruits Parkin. This event may induce autophagy by an ubiquitin-mediated mechanism. Parkin can also be phosphorylated by c-Abl increasing oxidative stress and promoting neuronal cell death.

Here we discuss Parkinson’s disease as a model system for the complex network induced by mitochondrial and nuclear DNA damage and oxidative stress. This disease has obtained in recent years a lot of interest (Figure 4). This interest is partially due to the high occurrence of PD, affecting more than 1% of the population over 65 years old. The major percentage of these cases is identified as sporadic PD and only a small percentage is considered familial PD [100].

Several causes can lead to sporadic PD insurgence such as the excessive exposure to heavy metals, pesticides or other toxic compounds and oxidative stress [101–103]. However, the most frequent causes of PD are mutations of the Parkin gene that encodes for an E3 ubiquitin ligase involved in an ubiquitin-mediated degradation pathway. Mutations in this gene cause an alteration of E3 ubiquitin ligase activity leading to an abnormal protein aggregation, one of the principal clinical features of PD [104–107].

Since Parkinson’s disease is characterized by various symptoms linked to different stages of its progression, the identification of this pathology at an early stage is not easy. In fact, the first symptoms are similar to normal aging progression such as rheumatism, fatigue, and depression along with sleep disturbance or loss of elasticity [108]. Only when the disease is in an advanced stage, most typical symptoms like motor loss and cognitive dysfunction become evident [100,107]. Four of the most typical motor symptoms of PD can be considered tremor at rest (the most common symptom of PD regarding principally the hands but also legs, lips jaw and chin and sometimes neck and voice) [109], rigidity (characterized by a reduction of normal flexion, extension or rotation of a limb) [110], bradikinesia (reduced capacity to plan and performs movements) [111], postural instability (loss of postural reflex) [112]. However, non-motor symptoms can also occur during PD progression; for example sleep disorders, neuropsychiatric disturbances (*i.e.*, dementia and compulsive behavior) and failure of autonomic function [113]. Disease progression is tightly linked to histopathological features of PD. PD is characterized by a loss of dopaminergic neurons in a specific area of *Substantia Nigra* (SN) accompanied by the formation of Lewy Bodies (LBs) and Lewy Neurites (LNs) that are intracellular inclusions principally constituted by α-synuclein (α-syn), a small protein expressed in SN, cerebellum, hippocampus and neocortex. Although it remains poorly resolved, α-syn can contribute to neural degeneration through a possible mechanism involving mitochondria. Several studies indicate that transgenic mice overexpressing wild type or mutant α-syn show abnormal mitochondrial morphologies [114]. The *N*-terminal membrane-binding domain of α-syn specifically binds to the membrane of mitochondria rather than to other organelles. In return, α-syn binding causes mitochondrial fragmentation. The amount of α-syn localized into mitochondria of SN neurons increases dramatically in PD [115]. Some authors have proposed another possible mechanism to enhance α-syn aggregation. This occurs through the interaction of α-syn with mitochondrial complex IV enzyme, cytochrome C oxidase (COX) leading to mitochondrial dysfunction and neuronal degeneration [116].

The LBs distribution is the base of Braak’s theory, which individuates six stages in PD progression. In the first three stages, there are no evident symptoms; particularly, in the first step where α-syn inclusions are present outside of SN, whereas in the second step LBs and LNs start to be deposed in the medulla oblongata. In stage three; α-syn deposits are present also in midbrain, basal forebrain and in a small part of SN. In stage four, the loss of neurons become evident in SN and in the cerebral cortex. In the last two stages α-syn inclusions invade both SN and neocortex and motor and cognitive dysfunctions occur [117].

### 6.2. Oxidative Stress and Mitochondrial Mutations/Dysfunction in Parkinson Disease

We have discussed above the role of oxidative stress for PD progression. Several post-mortem studies performed on individuals with Parkinson’s disease have shown an increased level of lipids, proteins and DNA oxidation and a decreased concentration of GSH. In these studies, enrichment of autophagosomes-like structures was observed [10]. Moreover, a loss of function of genes that encode for proteins involved in autophagy modulation and mitochondrial function has been described in PD. Accordingly, mitochondrial dysfunction seems to be implicated in PD insurgence [118]. Moreover, it has been indicated that mutations in the kinase PINK1 (PTEN induced putative kinase 1) and in Parkin are both implicated in mitochondrial quality control leading to development of autosomal recessive PD [106,119]. PINK1 is present in different districts of the brain, in particular in *substantia nigra*, hippocampus and Purkinje cells of cerebellum. PINK1 has a mitochondrial signal motif in the *N*-terminal domain and a *C*-terminal autoregulatory region. In healthy mitochondria, PINK1 is localized in the inner membrane and is degraded by the protease PARL (Presenilin Associated, Rhomboid-Like) [120]. Several studies demonstrate that PINK1 is involved in mitochondrial metabolism and dynamics, protein degradation ubiquitin-mediated and oxidative stress [121,122]. The role of PINK1 in PD progression is supported by the fact that PINK1 co-localizes with LBs [123]. Moreover, mice lacking PINK1 have typical symptoms of Parkinson’s disease including mitochondrial impairment of dopaminergic neurons [124]. Compelling evidence indicates that mutation of PINK1 is one of the principal causes of PD insurgence [125]. Parkin is another protein implicated in the pathogenesis of different neurodegenerative diseases and, particularly, in PD. Parkin has a *N*-terminal ubiquitin-like domain and a *C*-terminal RING box region with an E3 ubiquitin ligase activity [126]. Parkin is an important player in controlling the enrichment of protein aggregates. Albeit Parkin can reduce ROS production, the overexpression of mutant Parkin is linked to increased ROS generation. Parkin is associated with mitochondrial DNA. This gives a possible explanation of its protective role against oxidative stress. Post mortem studies performed in subjects affected by PD, demonstrate that Parkin colocalizes with LBs indicating an association with PINK1 and PD progression [127]. In damaged mitochondria, PINK1 translocates to the outer membrane, where it recruits E3 ligase Parkin from the cytosol. This induces the ubiquitination of outer membrane proteins ending in mitochondrial autophagy (mitophagy). In flies, Parkin accumulation and autophagy induction can cause an enrichment of impaired mitochondria in dopaminergic neurons [128]. In return, this generates an excessive amount of ROS. A reduced activity of mitochondrial complex I and its inhibitors in *Substantia Nigra* of individuals affected by PD has been recently investigated. Other post-mortem studies performed in *Substantia Nigra pars compacta* (SNpc) of PD patients, indicate an increase of oxidative stress related to dopamine metabolism due to oxidation of dopamine that can generate ROS like H_2_O_2_, which reacts with Fe^2+^ forming the reactive •OH by Fenton’s reaction. A consequence of these events is an alteration of the oxidative defense system leading to a reduced concentration of GSH and an increased level of GSSG. Loss of GSH is linked to a reduction of mitochondrial complex I activity in the SNpc, suggesting that decreased GSH is an early event after oxidative stress, ending later on in degeneration of dopaminergic neurons in idiopathic PD [10,73,74,91].

All the evidence observed in dopaminergic cells and compiled above regarding GSH depletion, mitochondrial complex I activity impairment, and increment of iron level in the context of increased oxidative stress can partially explain the dopaminergic cell death typical in PD. At this purpose, a theory has been postulated according to which in SNpc the level of oxidative stress is low in the physiological condition, but after some insults (for example due to toxic compounds or genetic mutations) the products of oxidative stress are increased. This situation, together with a reduction of GSH level, can create an alteration in the normal protein degradation pathway because of the hurdles for the proteasome to recognize and remove the oxidized proteins. The consequent impairment of protein clearance, generally accompanied by aggregate formation, eventually can lead to cell death [129]. Moreover, studies conducted in transgenic mice with mito-Pstl (mitochondria-targeted restriction enzyme that induces DSB in the mtDNA, leading to reduced oxidative phosphorylation, OXPHOS) indicate the important role of mtDNA and its role in cell death during PD [130]. Furthermore, a recent work investigated the relationship between the transcription factor p73 and tyrosine hydroxylase (a fundamental enzyme involved in dopamine synthesis) concluding that p73 can regulate the levels of tyrosine hydroxylase contributing, consequently to protection against PD [131].

On the other hand, studies conducted *in vitro* and *in vivo* indicate an association between c-Abl and Parkin. Compelling evidence indicates that pharmacological inhibition of c-Abl with STI-571 enhances E3 ligase activity of Parkin. Indeed, c-Abl phosphorylates E3 ligase Parkin on Y143, this induces the accumulation of Aminoacyl tRNA synthetase complex-interacting multifunctional protein 2 (AIMP2) and Fructose-1,6-bisphosphatase 1 (FBP-1). The latter are two toxic substrates of Parkin detected in the striatum. In this manner c-Abl can induce an alternative oxidative stress pathway inhibiting the ubiquitin-mediated pathway by Parkin and promote the accumulation of misfolded protein and toxic substrates (*i.e*., AIMP2 and FBP-1). Moreover, c-Abl activity seems to have a role in PD development also by regulating the activation of PKCδ, as shown in cell culture models of PD. Indeed, PKCδ (Protein kinase C, delta) is activated upon phosphorylation on Y311 by c-Abl, and this modification leads to cell death [89,132]. In addition, c-Abl activity can promote neuronal cell death induced by oxidative stress activating the Mammalian ste 20 like kinase (MST1) [133].

## 7. Conclusions

Parkinson’s disease is the second neuronal disorder, after Alzheimer’s disease (AD), afflicting people over 65 years of age. Despite extensive studies, there are no conclusive remarks regarding this pathology. As mentioned above only post-mortem studies are available for the investigation of PD. Nevertheless, recent reports have shown a very complex network of events underlying the insurgence and progression of PD [107,134]. An emerging theme is that persistent oxidative stress is at the basis of PD. This involves the active participation of mitochondria and of several proteins, such as c-Abl or Gluthatione *S*-tranferase in the signaling network underlying neuronal degeneration. Neuronal protection from oxidative stress represents an efficacious strategy against neurodegenerative diseases. While several studies pursuing some combined strategies have been reported, much remains to be done. Mapping the interplay between the different players involved in oxidative stress and DNA damage repair (both in the nucleus and mitochondria), is fundamental to understanding the disease progression and with this, to uncover new opportunities for effective therapeutic strategies.

## Abbreviations

AD: Alzheimer’s Disease
BER: Base Excision Repair
c-Abl: Abelson tyrosine kinase
CDK5: Cyclin-Dependent kinase 5
DSB: Double Strand Break
HR: Homologous Recombination
JNK: c-Jun N-terminal kinase
LBs: Lewy Bodies
LNs: Lewy Neurites
mtDNA: mitochondrial DNA
nDNA: nuclear DNA
NER: Nucleotide Excision Repair
NHEJ: Non Homologous End Joins
PD: Parkinson’s Disease
RNS: Reactive Nitrogen Species
ROS: Reactive Oxidative Species
SN: Substantia Nigra
SSB: Single Strand Break
α-syn: α-synuclein
